# Restrictive vs Liberal Blood Transfusion for Acute Upper Gastrointestinal Bleeding: Rationale and Protocol for a Cluster Randomized Feasibility Trial

**DOI:** 10.1016/j.tmrv.2013.04.001

**Published:** 2013-07

**Authors:** Vipul Jairath, Brennan C. Kahan, Alasdair Gray, Caroline J. Doré, Ana Mora, Claire Dyer, Elizabeth A. Stokes, Charlotte Llewelyn, Adam A. Bailey, Helen Dallal, Simon M. Everett, Martin W. James, Adrian J. Stanley, Nicholas Church, Melanie Darwent, John Greenaway, Ivan Le Jeune, Ian Reckless, Helen E. Campbell, Sarah Meredith, Kelvin R. Palmer, Richard F.A. Logan, Simon P.L. Travis, Timothy S. Walsh, Michael F. Murphy

**Affiliations:** a NHS Blood and Transplant, Oxford, United Kingdom; b Translational Gastroenterology Unit, John Radcliffe Hospital, Oxford, United Kingdom; c MRC Clinical Trials Unit, London, United Kingdom; d Department of Emergency Medicine, Royal Infirmary of Edinburgh, Edinburgh, United Kingdom; e NHS Blood and Transplant Clinical Studies Unit, Cambridge, United Kingdom; f Health Economics Research Centre, University of Oxford, United Kingdom; g Department of Gastroenterology, James Cook University Hospital, Middlesbrough, United Kingdom; h Department of Gastroenterology, St James’s University Hospital, Leeds, United Kingdom; i Nottingham Digestive Diseases NIHR Biomedical Research Unit, Nottingham, United Kingdom; j Gastrointestinal Unit, Glasgow Royal Infirmary, Glasgow, United Kingdom; k Department of Gastroenterology, Royal Infirmary of Edinburgh, Edinburgh, United Kingdom; l Department of Emergency Medicine, Oxford University Hospitals NHS Trust, Oxford, United Kingdom; m Department of Research and Education in Emergency Medicine, Acute Medicine, and Trauma, Nottingham University Hospitals NHS Trust, Nottingham, United Kingdom; n Department of Acute General Medicine, Oxford University Hospitals NHS Trust, Oxford, United Kingdom; o Western General Hospital, Edinburgh, United Kingdom; p Division of Epidemiology and Public Health and Nottingham Digestive Disease Centre, University of Nottingham, Nottinghamshire, United Kingdom; q Centre for Inflammation Research, Queen’s Medical Research Institute, University of Edinburgh, Edinburgh, United Kingdom; r Transfusion Medicine, Oxford University Hospitals NHS Trust, Oxford, United Kingdom; s University of Oxford, Oxford, United Kingdom

## Abstract

Acute upper gastrointestinal bleeding (AUGIB) is the commonest reason for hospitalization with hemorrhage in the UK and the leading indication for transfusion of red blood cells (RBCs). Observational studies suggest an association between more liberal RBC transfusion and adverse patient outcomes, and a recent randomised trial reported increased further bleeding and mortality with a liberal transfusion policy. TRIGGER (Transfusion in Gastrointestinal Bleeding) is a pragmatic, cluster randomized trial which aims to evaluate the feasibility and safety of implementing a restrictive versus liberal RBC transfusion policy in adult patients admitted with AUGIB. The trial will take place in 6 UK hospitals, and each centre will be randomly allocated to a transfusion policy. Clinicians throughout each hospital will manage all eligible patients according to the transfusion policy for the 6-month trial recruitment period. In the restrictive centers, patients become eligible for RBC transfusion when their hemoglobin is < 8 g/dL. In the liberal centers patients become eligible for transfusion once their hemoglobin is < 10 g/dL. All clinicians will have the discretion to transfuse outside of the policy but will be asked to document the reasons for doing so. Feasibility outcome measures include protocol adherence, recruitment rate, and evidence of selection bias. Clinical outcome measures include further bleeding, mortality, thromboembolic events, and infections. Quality of life will be measured using the EuroQol EQ-5D at day 28, and the costs associated with hospitalization for AUGIB in the UK will be estimated. Consent will be sought from participants or their representatives according to patient capacity for use of routine hospital data and day 28 follow up. The study has ethical approval for conduct in England and Scotland. Results will be analysed according to a pre-defined statistical analysis plan and disseminated in peer reviewed publications to relevant stakeholders. The results of this study will inform the feasibility and design of a phase III randomized trial.

ACUTE UPPER GASTROINTESTINAL bleeding (AUGIB) is the commonest reason for emergency hospital admission with a gastrointestinal disorder in the UK with an annual incidence of 50 to 150/100,000 adults [1]. It is the leading indication for red blood cell (RBC) transfusion, accounting for 14% of all RBCs transfused in England [2]. The purpose of RBC transfusion following AUGIB is to restore regional and global oxygen delivery. RBC transfusion is life-saving in exsanguinating patients, but most patients presenting with AUGIB do not have major hemorrhage, or features of hemodynamic compromise. In the 2007 UK audit of “AUGIB and the Use of Blood”, 62% of patients had no features of hemodynamic shock [3]. Most cases of AUGIB, regardless of etiology, will cease without the need for intervention. Many studies support this notion in that the rates of endoscopic intervention following AUGIB are in the order of 20-30% [4,5]. In most cases, RBCs are transfused because the hemoglobin (Hb) concentration has fallen below a threshold at which the physician believes the risks of anemia to outweigh the risks of transfusion. This perception of the appropriate threshold for transfusion is subjective but is likely to be influenced by multiple factors including the desire to have a “safe” Hb level in the event of re-bleeding, to reduce symptoms of anemia after bleeding has arrested, or by patient- and clinician-related factors. Hence, there is considerable practice variation with respect to RBC transfusion following AUGIB, with rates of transfusion ranging from 23% to 84% across 208 hospitals in the UK [3].

In other critically ill patient cohorts, a more liberal approach to transfusion has been associated with adverse clinical outcomes [6]. Two large observational studies have indicated a strong association between RBC transfusion after AUGIB and the risk of further bleeding, with a trend towards increased mortality, after adjustment for confounders [7,8]. These observations have now been supported by a recently published randomized trial in which rates of mortality and further bleeding were greater in the liberal transfusion arm [9]. Given the existing uncertainty and variation in transfusion practice for AUGIB and signals of harm associated with RBC transfusion, it is vital that the evidence base to inform the safe and effective use of RBCs is improved.

The purpose of this article is to describe the rationale and protocol for a cluster randomized controlled feasibility trial in the UK (TRIGGER—Transfusion in Gastrointestinal Bleeding) which aims to improve the evidence base for RBC transfusion in AUGIB. We also summarize the existing evidence and program of preliminary work, which has justified the need for and informed the design of the TRIGGER trial.

## Summary of Existing Evidence

### Evidence for Safe Transfusion Thresholds

Healthy humans can tolerate Hb levels as low as 5 g/dL without adverse consequences [10] and levels as low as 7 g/dL are safely tolerated in non-cardiac surgery, intensive care, and trauma patients [11]. However 13% of patients presenting with AUGIB in the UK have ischemic heart disease [3], raising concerns about the safe level of anemia in these patients. There are limited data indicating that subjects with coronary artery disease may be able to tolerate moderate normovolemic hemodilution well [12,13]. Lowering the Hb threshold for transfusion in patients undergoing coronary artery bypass surgery from 9 to 8g/dL [14], or from a hematocrit of 30% to 24%, did not result in higher morbidity or mortality [15]. The TRICC trial, a study comparing restrictive versus liberal RBC transfusion in intensive care patients, found no difference in mortality through use of a restrictive transfusion policy (Hb transfusion threshold < 7 g/dL) in a subgroup of 257 patients with known ischemic heart disease [16]. A systematic review of ten randomised controlled trials (RCTs) comparing clinical outcomes in restrictive versus liberal transfusion triggers concluded that a restrictive approach led to a 42% reduction in the probability of receiving transfusions with no effect on mortality, rates of cardiac events, morbidity, or length of hospital stay [11,17].

### Current Guidelines for RBC Transfusion in AUGIB

Current RBC transfusion practices in AUGIB are based upon consensus opinion. British Society of Gastroenterology guidelines recommend RBC transfusion when the Hb is ≤ 10 g/dL [18], but a more recent international guideline advocates transfusion when the Hb is ≤ 7g/dL [19]. For those with AUGIB secondary to portal hypertension, guidelines recommend maintaining the Hb around 8g/dL [20], but not higher. Although some consensus guidelines have advised against the use of a specific Hb level as an RBC transfusion trigger, several studies, mainly in critical care and surgery, have shown that clinicians attribute considerable importance to the Hb in making transfusion decisions [21]. This may be a reflection of the lack of physiological end points with sufficient sensitivity and specificity to guide transfusion decisions, especially outside a critical care setting.

### Sources of Harm and Cost of RBC Transfusion

Over the past decade there has been a marked change in critical care and surgical transfusion practice with an increased appreciation of the potentially harmful effects of allogeneic blood transfusion. Research in intensive care [22], cardiac surgery [23], and coronary care [24] suggest associations between blood transfusion and adverse patient outcomes, including death, even after adjustment for known confounders. Potential sources of harm from RBCs are poorly understood [25]. Some, such as transfusion-transmitted infection, transfusion-related acute lung injury, and hemolytic reactions are well defined but rare; transfusion-associated circulatory overload is unlikely to be rare but rather under-reported. Other possible mechanisms of harm include (1) adverse effects resulting from changes to RBCs that occur during blood storage [26]; (2) pro-inflammatory effects of blood transfusion [27] and; (3) transfusion of donor leukocytes, although the implementation of universal pre-storage leukocyte-reduction of blood components has reduced this risk [28].

Specific to AUGIB, the mechanism by which transfusion may be associated with further bleeding is unclear. In those patients with cirrhosis and portal hypertension who bleed from varices, excessive RBC transfusion is likely to worsen portal hypertension through volume overload, although it is possible that these effects may also occur with excessive fluid infusion per se as opposed to a unique effect of RBCs. However, this does not explain the mechanism in the majority of UK presentations with AUGIB in whom bleeding is non-variceal in origin (approx 90%). One postulated mechanism is that transfusion could counteract the splanchnic vasoconstrictive response after hypovolemic hemorrhage, resulting in a rebound increase in splanchnic blood pressure which could impair the ability to form clots [9]. It is unlikely that RBC transfusion would adversely impact upon the coagulation cascade to impair hemostasis. The mechanism of increased mortality with more liberal transfusion is most likely mediated through an excess of further bleeding, since this event is a strong and independent predictor of death [29].

## Preliminary Work to Inform the TRIGGER Trial

### The 2007 UK National Audit of AUGIB and the Use of Blood

This study provided detailed, “real-life” outcome data from 6750 patients with AUGIB admitted across 208 UK hospitals over a 2-month period [3,30]. The study highlighted areas of significant variation in transfusion practice and inappropriate use of RBCs; for example, 15% of all RBCs were transfused to patients with a Hb > 10 g/dL, who were hemodynamically stable. There was substantial clinical uncertainty in transfusion practice in patients presenting with a Hb between 8.1–10 g/dL. In this Hb range 51% of patients received transfusion and 49% did not. When patients were then stratified by the absence/presence of hemodynamic abnormality, the variability persisted. This range represents a genuine area of clinical uncertainty, practice variation, and potential equipoise in the RBC transfusion management of AUGIB.

### Outcomes Following Early RBC Transfusion for Acute Upper Gastrointestinal Bleeding

The relationship between early RBC transfusion, further bleeding, and mortality following AUGIB in 4,441 patients from the national audit data-set was examined. After adjusting for the clinical Rockall score [31] and initial Hb, early RBC transfusion was associated with a 2-fold increased risk of further bleeding (OR, 2.26; 95% CI 1.76-2.90) and a 28% increase in mortality (OR 1.28; 95% CI 0.94-1.74) [7]. Remarkably similar results were observed in a Canadian study of 1677 cases of AUGIB [8]. These studies suggest that many patients presenting with a Hb of > 8 g/dL following AUGIB may not benefit from transfusion, and could be harmed. Whilst these results do not prove a causal relationship, they indicate that a randomized comparison of restrictive and liberal transfusion policies in AUGIB is justified to confirm or refute the findings of these observational studies.

### A National Survey of Clinicians

A survey of UK clinicians was conducted with the aim of characterizing and understanding attitudes towards transfusion practice in patients with AUGIB [32]. Clinicians were asked to select a Hb threshold at which they would ordinarily transfuse RBCs in 6 realistic scenarios of AUGIB, and 815 clinicians responded. The most important patient characteristic raising the transfusion threshold was the presence of ischemic heart disease. The overall responses suggested belief that restrictive use of RBCs is appropriate, but were discordant with the observed practice in the national audit highlighting the discrepancy between theory and practice.

### A Systematic Review of Randomized Controlled Trials

A Cochrane systematic review was conducted in 2008 and updated in 2010 [33]. Only 3 small randomized controlled trials investigating differing RBC transfusion strategies in patients with AUGIB (total of 93 patients across 3 trials) were identified and all had methodological deficiencies. Whilst there was a trend towards increased mortality and further bleeding in the transfusion arms of the combined studies, the small number of participants and the large volume of missing data limited the generalizability of the findings. These data further support the need for a definitive randomized controlled trial to establish the risks and benefits of RBC transfusion in this population. The publication of a large trial subsequent to this Cochrane review will lead to an update of the review and a more precise estimate of treatment effects [9].

## Patients and Methods

### Trial Objectives

The objective of the TRIGGER trial is to evaluate the feasibility and safety of implementing a restrictive versus a liberal RBC transfusion policy in adult patients admitted with AUGIB, in order to inform the design of a definitive phase III trial.

### Design and Setting

TRIGGER is a pragmatic, cluster randomized feasibility trial comparing 2 different RBC transfusion strategies in patients admitted with AUGIB. Each participating center will be randomly allocated to one of 2 transfusion policies, and all eligible patients in a centre will be treated according to the allocated transfusion policy. The trial will recruit in 6 academic centers in the UK. Both feasibility and clinical outcome measures will be collected. Feasibility measures include recruitment rate, adherence to the transfusion policy, the difference in Hb, and RBC exposure between the restrictive and liberal transfusion policies and evidence of selection bias. Clinical outcomes include further bleeding, mortality, need for therapeutic endoscopy, need for surgery or radiological intervention to control bleeding, thromboembolic, and ischemic events, infections, transfusion reactions, and an assessment of quality of life at day 28 (EuroQol EQ-5D). Data will also be gathered to identify factors leading to contravention of the transfusion policy, and to enhance estimates of the intraclass correlation coefficient (ICC) to inform the sample size calculation for a phase III cluster randomized trial. The full trial protocol is available on the dedicated trial Web site: http://www.trigger.nhsbt.nhs.uk/.

### Randomization of Clusters

Widespread consultation was conducted amongst clinical stakeholders in each potential center to ensure a willingness to be randomized to either transfusion policy and agreement to implement the allocated policy on a hospital wide level. Centers will be randomized using permuted blocks without stratification or matching, in order to ensure an equal number of centers are assigned to each transfusion policy. Recruitment of patients will operate for 6 months in total in each center.

### Eligibility Criteria

Eligibility criteria relate both to those of the cluster and to the individual trial participants. Clusters (ie, hospitals) are eligible to take part if they meet the following criteria:

• > 20 admissions with AUGIB per month
• 400 hospital beds
• Availability of 24 hour endoscopy and on-site access to intensive care and surgical support
• Willingness to be randomly allocated to a transfusion policy
• Institutional agreement to transfuse all eligible new admissions with AUGIB in accordance with the randomized transfusion policy

Participants are eligible to take part in the study if:

• They are aged ≥ 18 years presenting to hospital with AUGIB, defined as hematemesis or the passage of melena.

Participants are not eligible to take part in the study if:

• The responsible clinician considers there is a need for immediate RBC transfusion prior to obtaining or regardless of the initial Hb result due to severity of bleeding.
• They are an existing hospitalized patient who subsequently develops AUGIB.

No upper age limit has been set for patient level inclusion criteria. In the large UK audit of AUGIB, 28% of presentations were aged ≥ 80 years (1898/6750) and excluding this group would limit both the anticipated recruitment rate and generalizability of the trial findings. Whilst the assessment of severe bleeding is left to clinical judgment, additional pragmatic guidance include (but are not exclusive to) patients with features of hemodynamic shock (systolic BP < 100 mmHg and/or heart rate > 100 bpm) and receipt of RBCs within 2 hours of presentation, transfusion of emergency O-negative blood, or performance of the index endoscopy in an emergency clinical area due to severity of bleeding.

### Interventions

The trial will compare 2 different policies for RBC transfusion, “restrictive” and “liberal”. All eligible patients admitted to a participating site should be transfused in accordance with the randomized transfusion policy.

#### Restrictive Transfusion Policy (RBCs Transfused When Hb ≤ 8 g/dL)

If the Hb value is recorded as ≤ 8.0 g/dL, RBCs should be transfused with the aim of keeping the Hb range between 8.1 and 10.0 g/dL. Further RBC transfusions should only be administered if the Hb value decreases to a value of 8.0g/dL or less.

#### Liberal Transfusion Policy (RBCs Transfused When Hb ≤ 10 g/dL)

If the Hb value is recorded as ≤ 10.0 g/dL, RBCs should be transfused with the aim of keeping the Hb range between 10.1 and 12.0 g/dL. Further RBC transfusions should only be administered if the Hb value decreases to a value of 10.0 g/dL or less.

In both intervention arms the number of RBC units transfused and the timing of repeat Hb measurements will be decided by the caring clinician. Any transfusion(s) required as part of the policy should be administered within 24 hours of obtaining the Hb result.

#### Clinician Discretion to Transfuse in Contravention of the Allocated Policy

All clinicians will have the discretion to transfuse, or not to transfuse, in contravention of the allocated policy for any patient admitted with AUGIB. Any clinician who deviates from the policy will be asked to provide the reason(s) for doing so on a Case Report Form. This is not designed to challenge the clinician’s judgment, but rather to identify patient subgroups, which may not be feasible to recruit into the planned phase III trial. Possible reasons for additional transfusions outside of the protocol could include periods of further bleeding or hemodynamic instability.

### Outcomes

The study will collect a range of feasibility outcome measures and clinical outcome measures.

Feasibility outcome measures for each treatment arm include:

• The proportion of eligible patients who provide consent.
• The proportion of patients who are ineligible due to need for immediate transfusion.
• Protocol adherence, which includes:
1. Overall adherence: the proportion of Hb counts for each patient where no deviation occurred. This will be averaged across all patients.
2. Adherence per Hb count: the overall proportion of Hb counts where no deviation occurred.
3. Adherence per patient: the proportion of enrolled patients for whom no transfusion deviation occurred.
• Selection bias: As the study is open-label, all clinicians will be aware of their hospital’s transfusion policy, resulting in the possibility of selection bias. The following outcomes will be compared between the 2 interventional arms in order to investigate this:
  o Age, shock, Hb at baseline, clinical Rockall score, Blatchford score, and the number of major comorbidities for consented patients
  o The difference in the baseline Hb, Rockall and Blatchford scores of consented vs. those not consented
• The number of RBC units transfused
• The proportion of patients receiving at least one RBC transfusion.
• The mean Hb over the first 7 days after admission
• The mean Hb over the entire study period (i.e. up to discharge/death/28 days)
• The mean Hb on discharge (the last recorded Hb prior to discharge will be used)

Clinical outcome measures include:

• Further bleeding up to day 28: further bleeding is a composite outcome that includes persistent bleeding and recurrent bleeding. Recurrent bleeding should initially be suspected in the event of any combination of the following: fresh hematemesis, continuous melena, or aspiration of fresh blood from a nasogastric tube, with a pulse rate of > 100 bpm, a fall in systolic blood pressure of > 30 mm Hg or a drop in Hb of > 2g/dL in the preceding 24 hours.
• Further bleeding up to hospital discharge
• All-cause mortality up to day 28.
• All-cause mortality up to hospital discharge
• Therapeutic intervention at the index endoscopy
• Surgical or radiological intervention to control bleeding up to death/discharge
• Proportion of patients experiencing the composite end point of thromboembolic and ischemic events up to Day 28. This is composed of myocardial infarction, stroke, pulmonary embolus, DVT, and acute kidney injury. Each component will also be assessed individually.
• Proportion of patients experiencing the composite end point of thromboembolic and ischemic events up to hospital discharge
• Acute transfusion reactions up to death/discharge. This is defined as a reaction occurring at any time up to 24 hours following a transfusion of a blood component.
• Infection up to day 28. This is defined as any infection necessitating a prescription for antibiotic treatment for a minimum of 5 days, provided the prescription is received before or on day 28
• Infection up to hospital discharge
• Length of hospital stay
• Health related quality of life at Day 28 (using EuroQol EQ-5D).
• Serious adverse events up to day 28.

### Participant Screening and Selection

Potential trial participants will be identified from Emergency Departments (ED) or Medical Admissions Units (MAU) from September 2012 until February 2013. All eligible patients will be managed in accordance with the hospital’s allocated transfusion policy, from presentation up to discharge. This will be facilitated through extensive education of clinical staff in acute admission areas, display of treatment algorithms in relevant clinical areas and a simple flagging system in blood banks to remind doctors of the hospital’s transfusion policy whenever a transfusion request for AUGIB occurs. The trial research nurse/member of the research team will screen admission lists daily and will be encouraged to have a strong presence in the emergency admissions area(s) to ensure admissions with AUGIB are identified daily. All eligible patients will be approached as soon as possible after presentation with AUGIB to obtain informed consent. All patients who provide written informed consent for data collection and follow-up at Day 28 will count as enrolments to the study. A schema of enrolment procedures is outlined in Figure.

### Consent and Ethical Considerations

Cluster randomized trials (CRTs) pose unique ethical challenges [34]. Consent may be sought at a number of levels in CRTs including from a “guardian” of the cluster for randomization of the cluster to the intervention, and/or consent from individual participants [35,36]. The consent process for this study was carefully considered in consultation with clinicians and patients. Consent will be sought from relevant clinical stakeholders at each center to enable the hospital to be randomized to the transfusion policy, such that all eligible patients admitted with AUGIB are treated with that policy for the duration of trial. Individual eligible participants will be approached as soon as possible after hospital presentation, if appropriate to his/her clinical condition, to consent for use of their routine hospital data and follow-up at day 28.

It is anticipated that the vast majority of patients will have capacity to provide consent. However, some patients may lack capacity to provide consent or have fluctuating capacity; in these cases guidance is provided by the Mental Capacity Act (England and NI; 2005) and Adults with Incapacity Act (Scotland; 2000).

### Consumer Involvement

We formed a TRIGGER trial focus group comprising members of the Patient Panel from the John Radcliffe Hospital, Oxford. Some members of this group had suffered from AUGIB and had received blood transfusions in the past. We discussed with the panel the ethical issues involved in the consent process and the complexities involved in cluster randomization. Two face-to-face focus groups with 9 members of the panel were conducted and involved an educational seminar about AUGIB and clinical trials.

We used a mixture of both qualitative and quantitative methods of feedback and all participants completed an anonymous questionnaire. All participants supported (as indicated both in the discussion and anonymous feedback forms) the consent processes outlined. Specifically they unanimously supported the notion of automatic treatment of eligible patients with the hospital’s allocated transfusion policy without the need to obtain patient consent for the intervention, given the existing variation in transfusion practice we have demonstrated throughout the UK and the fact that both policies are within current practice. However, they stressed that all clinicians involved in the patient’s care should have the discretion to transfuse in contravention of the policy if they wish to do so, such that patient safety is not compromised. Members of the panel also helped to inform the content of the information sheets and consent forms. A similar process was conducted with a patient group through Age UK and the summaries of these meetings helped to inform our ethics application.

### Sample Size

As this is a feasibility study, no formal sample size calculation was undertaken to determine the number of patients required based upon a primary end point. Using information on the number of admissions with AUGIB at each participating centre, we estimated 1062 eligible patients would present over the course of 6 months. Assuming an 80% consent rate, this would give 849 patients enrolled in total.

This feasibility study will also help to inform the sample size calculation for the definitive phase III trial by allowing estimates of the rate and ICC for further bleeding, which is likely to form the primary outcome of the phase III trial.

TRIGGER is not powered on the end-points of further bleeding or mortality. However safety is being monitored by review of serious adverse events by an independent data monitoring committee. Any large imbalances between treatments that suggest harm would influence the feasibility of a larger follow on trial, as is the case in all early phase studies.

### Data Collection

#### Screening Log

A daily screening log of all patients admitted with AUGIB will record the total number of admissions, the number meeting eligibility criteria and eligible patients enrolled in each hospital. Anonymized, baseline clinical characteristics for each patient will be recorded.

#### Baseline Characteristics

Baseline clinical characteristics will be obtained from routine patient records and will include age, gender, presenting symptoms, routine physiological parameters, Rockall and Blatchford score [37] and use of anti-platelets/anticoagulants.

#### Laboratory and Transfusion Data

The date and time of all Hb measurements will be recorded as well as the first coagulation screen, liver function test, and biochemistry profile. All RBC transfusion episodes will be recorded including the Hb level prior to transfusion and the total number of RBC units transfused. The number of other blood components administered including fresh frozen plasma and platelets will be recorded.

#### Endoscopic and Pharmacological Data

Endoscopic data will include date and time of endoscopy, endoscopic diagnosis, stigmata of hemorrhage, and nature of therapeutic procedure. Pharmacological data will include use of proton pump inhibitors before and after endoscopy and the use of antibiotics and vasopressors for patients with variceal hemorrhage.

#### Day 28 Follow-Up

Day 28 follow-up will be conducted by telephone to assess a list of pre-defined clinical outcomes, quality of life (QoL), and resource use details. The patient’s primary care physician will first be telephoned to ascertain survival status before calling the patient. A telephone version of the EQ-5D questionnaire will be used to assess QoL. For patients who are still in hospital at Day 28 this will be conducted face-to-face.

### Data Management

All data will be recorded on paper case report forms. Centers will send copies of completed case report forms to the data manager at the NHS Blood and Transplant Clinical Studies Unit, where they will be entered onto a bespoke trial database designed using the MACRO v3.2 clinical trial software management system supplied by InferMed Limited.

### Data Analysis

All analyses will be described in full detail in a Statistical Analysis Plan, which will be finalized prior to data lock and analysis. Outcomes will be analyzed using cluster level summaries which will account for the correlation between patients in the same center. For feasibility outcomes, all enrolled patients will be included in the analysis. For clinical outcomes, the primary analysis will only include patients whose Hb dropped below 12 g/dL during the follow up period. This is to restrict the clinical analysis to those patients who are likely to have been affected by the transfusion policy. A cut-off of 12 g/dL was selected to ensure the majority of transfused patients will be included in the analysis. A secondary analysis of clinical outcomes will include all patients.

For the main trial, the primary outcome is likely to be further bleeding. The ICC for further bleeding estimated from this feasibility study will be imprecise, as it will be based on only 6 centers. In order to obtain a more precise estimate of the ICC on which to base the sample size for the main trial, the ICC from the feasibility study will be combined with the ICC obtained using observational data from the national audit of AUGIB.

### Health Economics

Health economics will be incorporated in to the feasibility trial in 2 ways. Firstly, the feasibility of gathering data required to facilitate a cost-effectiveness analysis (eg, resource use, costs, outcomes) will be examined to inform data collection for a phase III trial. Secondly, the feasibility of using these data within a cost-effectiveness model will be explored to identify parameters with the potential to be key drivers of cost-effectiveness, and hence requiring detailed measurement in the planned phase III trial. Within this feasibility trial, costs associated with hospitalization for AUGIB in the UK will be generated for the first time.

### Ethics

The trial received favorable ethical opinion from the NRES Committee South Central- Oxford C, REC reference 12/SC/0062 for conduct in England and by the Scotland A Research Ethics Committee for conduct in Scotland, REC reference 12/SS/0023. Site specific R&D department approvals will be obtained in each recruiting site in England and Scotland. The trial registration number is ISRCTN: 85757829 and the trial is registered on the NIHR Clinical Research Network (Study ID: 12078 Oral and Gastrointestinal, co-adopted by Blood, Injuries and Emergencies).

### Trial Management

The Trial Management Group will be responsible for the day to day conduct of the trial. An independent Trial Steering Committee (TSC) will provide overall supervision for the trial and provide advice to the Trial Management Group through its independent Chair. Other members of the TSC will include 2 independent clinicians and a lay representative. The ultimate decision for the continuation of the trial lies with the TSC. A core independent Data Monitoring Committee (IDMC) will monitor safety, progress, and quality of the trial. The composition of the committee is detailed in the study protocol, which is available on the trial website (http://www.nhsbt.nhs.uk/trigger/). The trial is being coordinated through the NHS Blood and Transplant Clinical Studies Unit/MRC Clinical Trials Unit.

## New Evidence Which Has Arisen During the Conduct of TRIGGER

At the time of submission of this manuscript, new evidence has arisen from a single centre RCT in a specialist gastrointestinal bleeding unit in Barcelona indicating benefit of a restrictive approach to transfusion in patients with AUGIB [9]. Over a 6-year period from 2003–2009 this RCT enrolled 39% (921/2372) of presentations with AUGIB into a trial of restrictive (eligible for transfusion when Hb < 7 g/dL to maintain Hb at 7–9 g/dL; n = 461) versus liberal (eligible for transfusion when Hb < 9 g/dL to maintain Hb at 9–11 g/dL; n = 460) RBC transfusion strategies. The rates of further bleeding were significantly lower in the restrictive transfusion arm (10% vs 16%) and 45 day mortality was also lower in the restrictive transfusion arm (5% vs 9%). The overall adverse event rate was higher in the liberal transfusion arm (48% vs 40%), the key differences being an increase in transfusion associated circulatory overload and transfusion reactions in the liberal arm.

Aside from the transfusion thresholds and randomization methodology, there are a number of important differences between TRIGGER and the Villanueva trial. Firstly, the patient population differs from the population which will be expected in TRIGGER. In particular: (1) 31% of patients in the Villanueva study had liver cirrhosis while this figure is likely to be < 10% in TRIGGER; (2) TRIGGER will include patients with ischemic heart disease and cardiovascular disease, stroke, and peripheral vasculopathy, which were notable exclusions in the Villanueva study. These are important to consider when extrapolating the findings of the results (ie, the trial’s external validity); (3) the Villanueva trial did not show any benefit for the restrictive policy in patients with peptic ulcer bleeding who are likely to form the largest group in TRIGGER, based upon UK epidemiology; and 4) the external validity of the Villanueva trial is limited as it arises from a single tertiary centre with strict protocols of care administered to all patients in the trial, which may not reflect routine care in most healthcare institutions e.g. the ability to provide therapeutic endoscopy within 6 hours for all patients no matter what time or day they are admitted, since the availability of such an intervention may in turn influence thresholds for transfusion.

The Villanueva trial was published after 75% of recruitment for TRIGGER was completed. The availability of this new external evidence had the potential to influence the conduct and methodology for the remainder of recruitment. All recruiting sites were contacted within 24 hours of publication of the Villanueva trial to discuss the trial findings and after local discussions they all expressed a willingness to continue recruitment to TRIGGER. Indeed, in view of the highlighted differences between the studies and the limited external validity of the Villanueva study, it was felt to be even more important to produce further evidence to confirm or refute the findings of the Villanueva trial. In addition we contacted the ethics committee, IDMC, and TSC following publication of the Villanueva study. The IDMC performed an unscheduled review of serious adverse events, mortality, and further bleeding. Based on the results of this unscheduled review, and in light of the differences between the 2 trials, they recommended continuation of TRIGGER. All independent members of the TSC recommended that we continue the trial for similar reasons. Whilst the Villanueva trial adds important new information to this subject area, further pragmatic trials are warranted and the design of TRIGGER will both help to inform this debate as well as address some of the limitations of the trial.

## Discussion

In the UK, an estimated 350,000 units of RBCs are administered annually to patients with AUGIB, making it the most common single indication for transfusion of RBCs. The direct costs are approximately £45.5 million for the blood alone, which does not take into account costs associated with administration, patient monitoring, and managing adverse events. The preliminary work leading up to this trial identified a lack of evidence informing the RBC transfusion management of AUGIB, and widely varying use of RBC transfusion amongst UK clinicians including evidence of liberal approaches to transfusion.

Two large observational studies found an independent association between RBC transfusion and adverse clinical outcome, notably an increased risk of further bleeding [7,8], and a recent randomised trial [9] reported an increase in further bleeding and mortality with a liberal transfusion policy. Further bleeding is of clinical importance since this one of the most important predictors of mortality, and further bleeding rates have not improved in longitudinal UK data over the past 15 years [29], despite advances in endoscopic therapy. Taken together, these observations support the need for an RCT comparing different RBC transfusion strategies in AUGIB in order to inform the safe and effective use of RBC transfusion.

Conducting an RCT of RBC transfusion strategies in patients with AUGIB is challenging. AUGIB is a medical emergency, where the majority of patients are admitted out of normal working hours and there is often a need for early administration of blood components. In the UK, patients are usually managed in several different clinical areas within hours of presentation, under the care of a broad range of clinicians from different teams (including Emergency Medicine, Acute Medicine, and Gastroenterology). With these challenges in mind, the TRIGGER trial has been designed to assess the feasibility and safety of implementing a restrictive versus liberal RBC transfusion policy in adult patients admitted with AUGIB.

The methodology of the trial was carefully considered after wide ranging consultation with frontline clinicians, clinical trialists, methodologists, and patient groups. Cluster randomization was deemed to be the most appropriate methodology to address some of the aforementioned challenges and in addition to avoid the high perceived risk of contamination between interventions if individual randomization was used. The feasibility end points of the trial will help to establish whether a broad range of clinicians can sufficiently adhere to a transfusion policy in order to justify a follow on phase III trial, and the clinical end points will help to inform the primary end point upon which to power the trial.

Whilst a more restrictive approach to transfusion has been demonstrated to be as safe [11], or in some circumstances, superior to a liberal transfusion policy [6], it is important to note that very few of these studies have been conducted in the setting of patients presenting to hospital with acute hemorrhage. Indeed, any history of bleeding was an exclusion criterion in a RCT of 2 differing RBC transfusion strategies in critically ill patients [6]. The transfusion thresholds chosen in the TRIGGER interventional arms are based on modeling using a large observational dataset of actual transfusion practice in the UK and are within the recommendations included in UK national and local clinical guidelines. Importantly, all clinicians taking part in the trial have the discretion to transfuse, or refuse to transfuse, outside of the policy that their hospital has been allocated by cluster randomization. The reasons for protocol violations are being recorded and these may help to further inform eligibility criteria for a phase III trial.

RBCs are a costly, finite, and scarce resource. Improving the evidence base for their safe and effective use is of major public health importance. The TRIGGER trial will begin to inform the evidence base for the leading indication for RBC transfusion in the UK. Uniquely, it will also provide an estimate of the costs associated with hospitalization for AUGIB in the UK, and assess health related quality of life at 28 days post presentation. The study will influence the wider debate about restrictive prescribing of blood products and the utilization of both blood conservation and avoidance strategies in other clinical settings. The results of the study will inform the feasibility and design of a larger phase III trial.

## Figures and Tables

**Fig:**
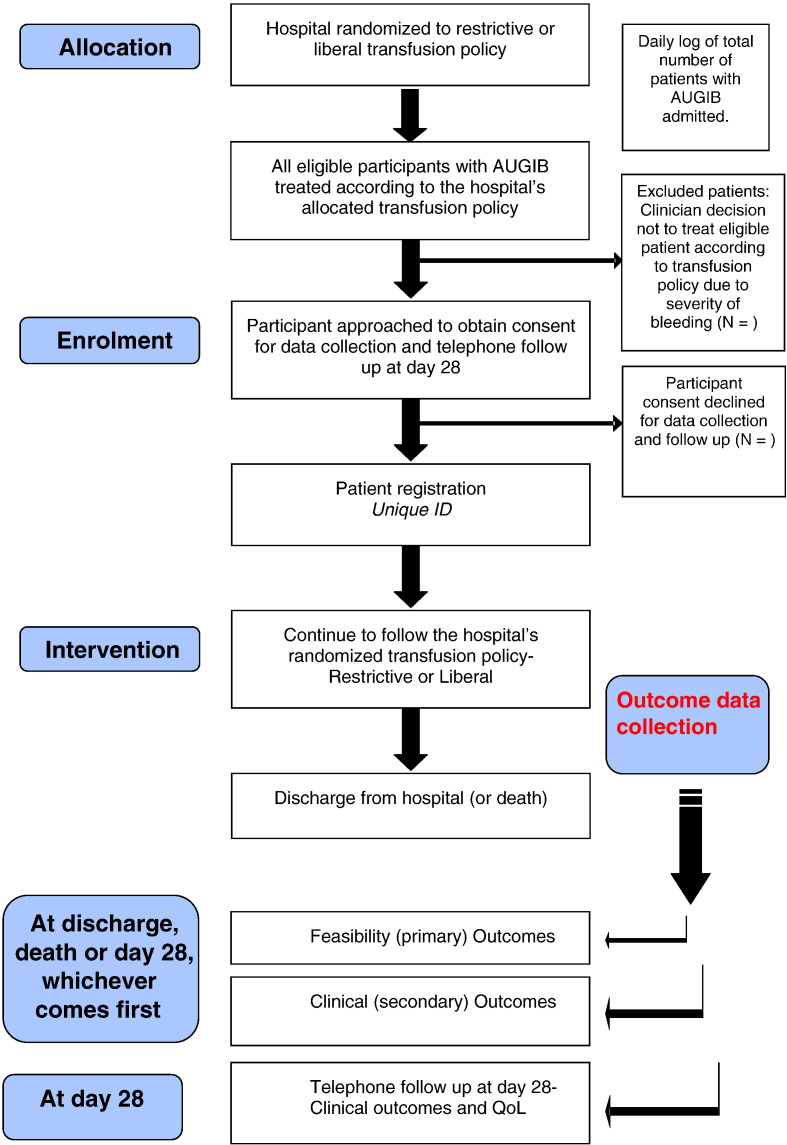
Trial schema.
